# Characterization of the SAM domain of the PKD-related protein ANKS6 and its interaction with ANKS3

**DOI:** 10.1186/1472-6807-14-17

**Published:** 2014-07-07

**Authors:** Catherine N Leettola, Mary Jane Knight, Duilio Cascio, Sigrid Hoffman, James U Bowie

**Affiliations:** 1 Department of Chemistry and Biochemistry, UCLA-DOE Institute of Genomics and Proteomics, Molecular Biology Institute, University of California, Los Angeles, Boyer Hall 611 Charles E. Young Dr. E, Los Angeles, California 90095-1570, USA; 2 Medical Research Centre, Klinikum Mannheim, University of Heidelberg, D-68167 Mannheim, Germany

**Keywords:** Polycystic kidney disease, Protein-protein interaction, Polymerization, Crystal structure

## Abstract

**Background:**

Autosomal dominant polycystic kidney disease (ADPKD) is the most common genetic disorder leading to end-stage renal failure in humans. In the PKD/Mhm(cy/+) rat model of ADPKD, the point mutation R823W in the sterile alpha motif (SAM) domain of the protein ANKS6 is responsible for disease. SAM domains are known protein-protein interaction domains, capable of binding each other to form polymers and heterodimers. Despite its physiological importance, little is known about the function of ANKS6 and how the R823W point mutation leads to PKD. Recent work has revealed that ANKS6 interacts with a related protein called ANKS3. Both ANKS6 and ANKS3 have a similar domain structure, with ankyrin repeats at the N-terminus and a SAM domain at the C-terminus.

**Results:**

The SAM domain of ANKS3 is identified as a direct binding partner of the ANKS6 SAM domain. We find that ANKS3-SAM polymerizes and ANKS6-SAM can bind to one end of the polymer. We present crystal structures of both the ANKS3-SAM polymer and the ANKS3-SAM/ANKS6-SAM complex, revealing the molecular details of their association. We also learn how the R823W mutation disrupts ANKS6 function by dramatically destabilizing the SAM domain such that the interaction with ANKS3-SAM is lost.

**Conclusions:**

ANKS3 is a direct interacting partner of ANKS6. By structurally and biochemically characterizing the interaction between the ANKS3 and ANKS6 SAM domains, our work provides a basis for future investigation of how the interaction between these proteins mediates kidney function.

## Background

Autosomal dominant polycystic kidney disease (ADPKD) is the most common inherited renal cystic disease, with a prevalence of approximately 1 in 1,000 individuals [1-3]. ADPKD is characterized by the progressive formation of fluid-filled cysts within the kidney which ultimately disrupt renal function, as well as various extrarenal manifestations [3,4]. Mutations in two genes, *PKD1* (polycystic kidney disease-1) and *PKD2* (polycystic kidney disease-2) account for approximately 85% and 15% of disease cases, respectively [2]. The occurrence of disease in patients lacking mutations in either of these genes suggests the involvement of other genetic loci, but this is still uncertain [5]. *PKD1* and *PKD2* encode the proteins polycystin-1 (PC1) and polycystin-2 (PC2), respectively. PC1 is a multidomain membrane receptor capable of binding and interacting with proteins, lipids, and carbohydrates and stimulating intracellular signaling pathways. PC2 is a membrane protein which acts as a Ca^2+^-permeable non-selective cation channel. PC1 and PC2 can interact, and in the process, modulate each other’s activity [2,6].

**Figure 1:**
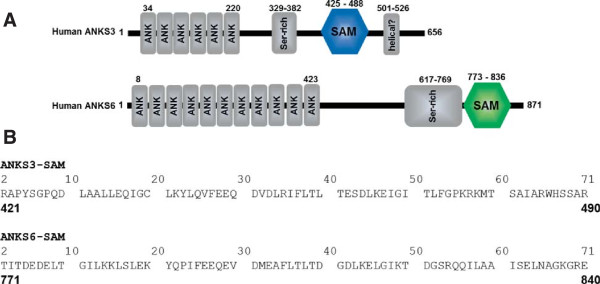
**The ANKS3 and ANKS6 proteins. A)** The domain structure of human ANKS3 and human ANKS6. ANK = ankyrin repeat, SAM = sterile alpha motif, Ser-rich = serine rich region, helical = potential coiled coil domain. **B)** The sequences of the human ANKS3-SAM [UniProt:Q6ZW76] and ANKS6-SAM domains [UniProt:Q68DC2] used in this work. The top numbering is the numbering used in the crystal structures. Each sequence contains an additional Ser at the N-terminus that is not shown. The numbers in bold below each sequence correspond to the numbering in the full length proteins.

Among the animal models of polycystic kidney disease, the PKD/Mhm(cy/+) rat recapitulates many of the hallmarks of human ADPKD [7-10]. The Cy mutation was found to be a missense mutation in the *ANKS6* gene, encoding ankyrin repeat and SAM-domain containing protein 6 (ANKS6) [11]. Human ANKS6 is an 871 amino acid protein containing 11 ankyrin repeats at its N-terminus and a SAM domain near its C-terminus (Figure 1A). The Cy mutation occurs in the SAM domain and generates an arginine to tryptophan mutation at amino acid 823 [11]. The R823W point mutation acts in a dominant-negative fashion, as evidenced by the PKD phenotype of transgenic rats over-expressing mutated *ANKS6*^(p.R823W)^[12].

Recently ANKS6 has also been implicated in human renal disease. In particular, the work Hoff and Halbritter et al. [13] has placed ANKS6 as a central node in a network of nephronophthisis (NPHP)-associated proteins, including direct binding between the ankyrin-repeat domain of ANKS6 and NEK8, a NimA (never in mitosis A)-related serine-threonine kinase which is mutated in nephronophthisis. Moreover, 8 individuals from 6 different families bearing homozygous mutations in *ANKS6* all presented with nephronophthisis, which is essentially an infantile or juvenile onset of PKD [13]. While several of the detected mutations localize to the ankyrin repeats, one generates a truncation within the SAM domain at Tyr790. Thus, the SAM domain has been shown in both rats and humans to be essential for normal ANKS6 function.

SAM domains consist of approximately 70 amino acids and adopt a globular structure generally containing a core of five α-helices [14,15]. Most SAM domains that have been characterized are protein-protein interaction modules that either self-associate [16-22], bind to other SAM-domain containing proteins [14,23-27], or bind other proteins altogether [14,28,29]. Some SAM domains have been found to bind RNA and lipids, however [30,31]. The protein-protein interactions of SAM domains are typically mediated by two distinct surfaces on the domain, termed the mid-loop (ML) and end-helix (EH) surfaces. SAMs can bind each other via their ML and EH surfaces, to generate open-ended polymers [16,18,19,24,32], closed oligomers [23,24], and heterodimers [24,27,33,34]. Through polymer formation and heterotypic interactions, SAM domains confer a diverse array of biological functions including gene regulation [18,24], enzyme localization [16,35], and scaffolding [19,20,36].

In spite of the important role of the ANKS6-SAM domain in cystic kidney disease, the function of ANKS6 remains unknown. Prior work using a proteomics screen of tandem affinity purified (TAP)-tagged NPHP-associated proteins found that ANKS6 and ankyrin repeat and SAM-domain containing protein 3 (ANKS3) are potential binding partners [13]. ANKS3 has a similar domain structure to ANKS6 including a C-terminal SAM domain (Figure 1A). Here we define the ANKS6/ANKS3 interaction by discovering that the ANKS6 SAM domain binds to the SAM domain of ANKS3. We show that ANKS3-SAM forms polymers and that ANKS6 binds to one end of these polymers. The R823W mutation was found to disrupt the structure of the ANKS6 SAM domain and negatively affects binding to ANKS3-SAM. Our results provide a structural explanation for the defect in PKD/Mhm(cy/+) rats and a potential new pathway to cystic disease via the SAM domain of ANKS3.

## Results

### negGFP native gel screen identifies ANKS3-SAM + ANKS6-SAM interaction

**Figure 2:**
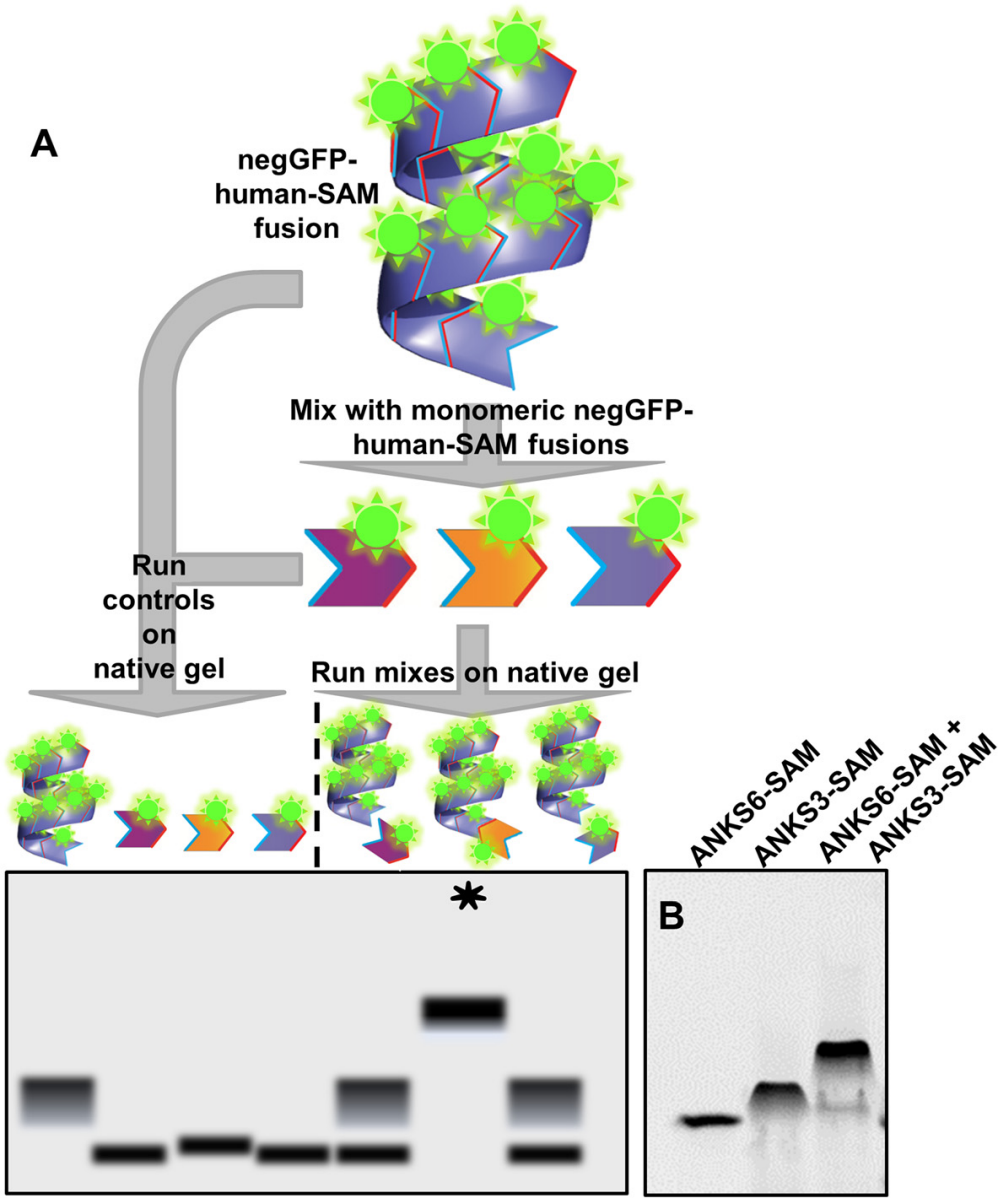
**ANKS3-SAM binds to ANKS6-SAM. A)** Schematic of the negGFP native gel binding assay. Lysate containing a negGFP-human-SAM fusion protein is mixed with lysate containing a negGFP fusion of a different human SAM domain. Each SAM domain contains an ML-surface (red) and an EH-surface (blue). Mixes and individual SAM controls are run on native gels and visualized by fluorescence. Novel hetero-SAM interactions appear as a new upshifted band (asterisk) in the gel schematic. **B)** negGFP fusions of ANKS6-SAM, ANKS3-SAM, and a 1:1 mix of ANKS6-SAM + ANKS3-SAM assayed by native gel electrophoresis. ANKS6-SAM appears monomeric, ANKS3-SAM appears weakly polymeric, and the appearance of a new distinct band upon mixing the proteins indicates an interaction.

To identify new human SAM domain hetero-interactions involving ANKS3, we employed a rapid screen for binding activity, outlined in Figure 2A. SAM domains were fused to an engineered green fluorescent protein modified to have a net charge of −30 (negGFP) [22,37]. The high charge on the negGFP effectively solubilizes even insoluble proteins, and leads to consistent migration of the fusion proteins towards the cathode on a native gel [22]. Binding between two negGFP fusion proteins is detected as the appearance of a new band with retarded migration on native gels. Because the fusions are fluorescent, assays can be performed using crude extracts. Thus, many different combinations of proteins can be rapidly screened using this technique.

Extract containing negGFP-ANKS3-SAM fusion protein was mixed with extracts containing negGFP-SAM fusions from 40 different human SAM domain-containing proteins (Additional file 1) and binding was tested by native gel electrophoresis. Among the human SAMs assayed, we detected a novel interaction between ANKS3-SAM and ANKS6-SAM (Figure 2B). Consistent with prior results, negGFP-ANKS6-SAM appears monomeric as it runs as a discrete band with migration similar to other monomeric SAM domains [22]. In contrast, the negGFP-ANKS3-SAM fusion runs as a more diffuse band with slightly slower migration, behavior that was observed previously and is typical of weakly polymeric SAM domains [22]. When negGFP-ANKS3-SAM and negGFP-ANKS6-SAM were mixed in a 1:1 ratio, the appearance of a new slower migrating species indicates that ANKS3-SAM and ANKS6-SAM bind to each other. We therefore decided to investigate the polymeric character of ANKS3-SAM and the novel ANKS3-SAM + ANKS6-SAM hetero-interaction further.

### ANKS3-SAM is polymeric

**Figure 3:**
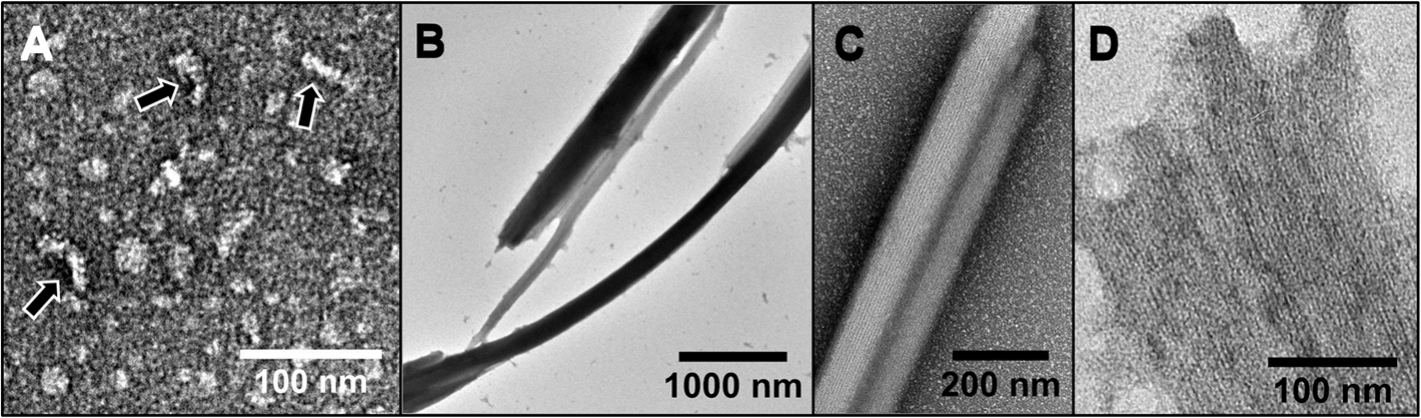
**ANKS3-SAM assembles into polymers. A)** Negative stain TEM of the negGFP-ANKS3-SAM fusion protein reveals short helical polymers (arrows). **B**-**D)** His_6_-tagged ANKS3-SAM (negGFP removed) precipitates as large fibrous sheets of polymers.

We first chose to characterize the ANKS3-SAM domain and investigate whether ANKS3-SAM forms a polymer. In a prior screen for SAM polymers, negGFP-ANKS3-SAM was observed by negative stain transmission electron microscopy (TEM) to form short polymeric structures [22]. We re-examined the negGFP-ANKS3-SAM fusion and obtained results consistent with earlier work, showing short polymers 11.4 ± 2.2 nm wide on average and varying in length from 20–90 nm, with an average length of approximately 36 nm (Figure 3A).

Since the addition of negGFP in the SAM fusion proteins introduces charge repulsion that weakens SAM domain interaction and polymerization, we next examined the ANKS3-SAM domain by itself. Without the negGFP fusion, ANKS3-SAM was much less soluble and precipitated after purification. We examined the precipitate by TEM and saw enormous sheets of polymers, some extending more than 1 μm long and 0.4 μm wide (Figure 3B-D). Individual polymers within the fiber-like sheets were approximately 4–6 nm wide. The ability of SAM domains to organize as sheets of polymers has been seen previously with the SAM domains of DGKδ and Shank3 [19,35], however it is unclear whether sheet formation is physiologically relevant for ANKS3-SAM.

### Mapping the ANKS3-SAM polymer

**Figure 4:**
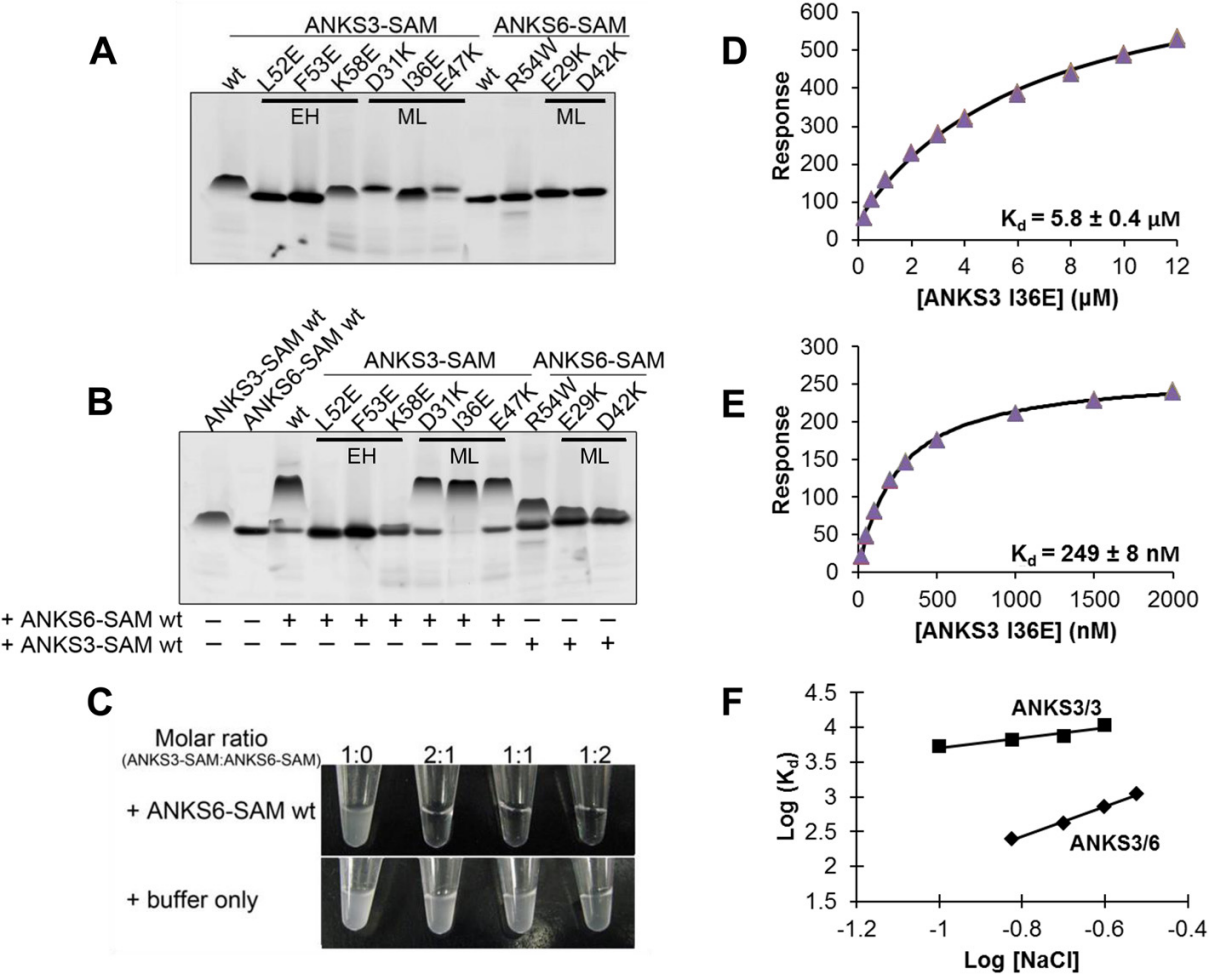
**Characterizing the ANKS3-SAM polymer interface and the ANKS3-SAM/ANKS6-SAM interaction interface. A)** The negGFP native gel screen identifies point mutations in both the EH- and ML-surfaces of ANKS3-SAM that result in a loss of polymeric character. Point mutations in ANKS6-SAM do not impact the native gel migration or monomeric character of this SAM domain. **B)** Mixing negGFP-ANKS3-SAM and negGFP-ANKS6-SAM mutants in 1:1 molar ratios reveals that the EH-surface of ANKS3-SAM binds the ML-surface of ANKS6-SAM. Point mutations on the EH-surface of ANKS3-SAM inhibit interaction with ANKS6-SAM whereas point mutations on the ML-surface of ANKS3-SAM do not affect the hetero-interaction. Point mutations on the ML-surface of ANKS6-SAM and the Cy mutation (R54W according to our numbering) inhibit interaction with ANKS3-SAM. **C)** ANKS3-SAM precipitate is resolubilized by the addition of ANKS6-SAM but not by the addition of buffer alone. **D)** The binding affinity of the native ANKS3-SAM polymer interface is measured by SPR (K_d_ = 5.8 ± 0.4 μM). Equilibrium binding measurements were performed in triplicate and fit to a 1:1 steady-state model. The calculated K_d_ is an approximation since tested analyte concentrations were insufficient to reach saturation. The error bars are smaller than the data points. **E)** The binding affinity of the native ANKS3-SAM/ANKS6-SAM interface is measured by SPR (K_d_ = 249 ± 8 nM). Equilibrium binding measurements were performed in triplicate and fit to a 1:1 steady-state model. The error bars are smaller than the data points. **F)** The binding between both ANKS3-SAM/ANKS3-SAM and ANKS3-SAM/ANKS6-SAM exhibits a salt dependency. The K_d_ of each interaction was determined by SPR at four different ionic strengths. The slope of the linear fit (~2 for ANKS3-SAM/ANKS6-SAM, ~1 for ANKS3-SAM/ANKS3-SAM) indicates that each interface is salt-dependent and employs ionic interactions.

To determine the interfaces of ANKS3-SAM responsible for polymer formation, we employed our negGFP binding assay to rapidly screen for point mutants that blocked polymerization. A similar approach was used in our previous study of the Caskin1 tandem SAM domains [20]. We targeted putative ML and EH surface residues to find those that yielded faster migration on a native gel. As shown in Figure 4A, the mutations D31K, I36E and E47K on the ML-surface and L52E, F53E and K58E on the EH-surface inhibited polymerization. Thus, ANKS3-SAM appears to form a polymer using an interface common to other SAM polymers [14].

The identification of monomeric ANKS3-SAM mutants allowed us to measure binding affinity between subunits using surface plasmon resonance (SPR). To measure affinity of the native interface, we immobilized an ANKS3-SAM EH-surface mutant, F53E, on an SPR chip and detected equilibrium binding to an ML-surface mutant, I36E. At 0.15 M NaCl, we observe hyperbolic binding with a K_d_ of 5.8 ± 0.4 μM (Figure 4D). As many of the mutations that reduce polymerization involve charged residues, we also examined the salt dependence of binding. As shown in Figure 4F, where the salt sensitivity of a protein-protein interaction is indicated by the slope of a log K_d_ versus log [salt] plot [38,39], the binding affinity is indeed strongly dependent on salt concentration.

### Structure of the ANKS3-SAM polymer

To better understand how the ANKS3-SAM domain forms polymers we sought a crystal structure of the polymer. Because the wild-type SAM domain is relatively insoluble, forming heterogeneous polymers, it cannot be crystallized directly. We therefore used a strategy that has proven successful for a number of other SAM domain polymers, where we attempt to crystallize SAM domains with mutations in the polymer interface [16,18,24]. The mutations weaken subunit association so that the protein remains soluble during purification but under the high concentrations required for crystallization, the polymer interface remains a favorable site for crystal contacts, thereby generating the polymer in the crystal.

**Figure 5:**
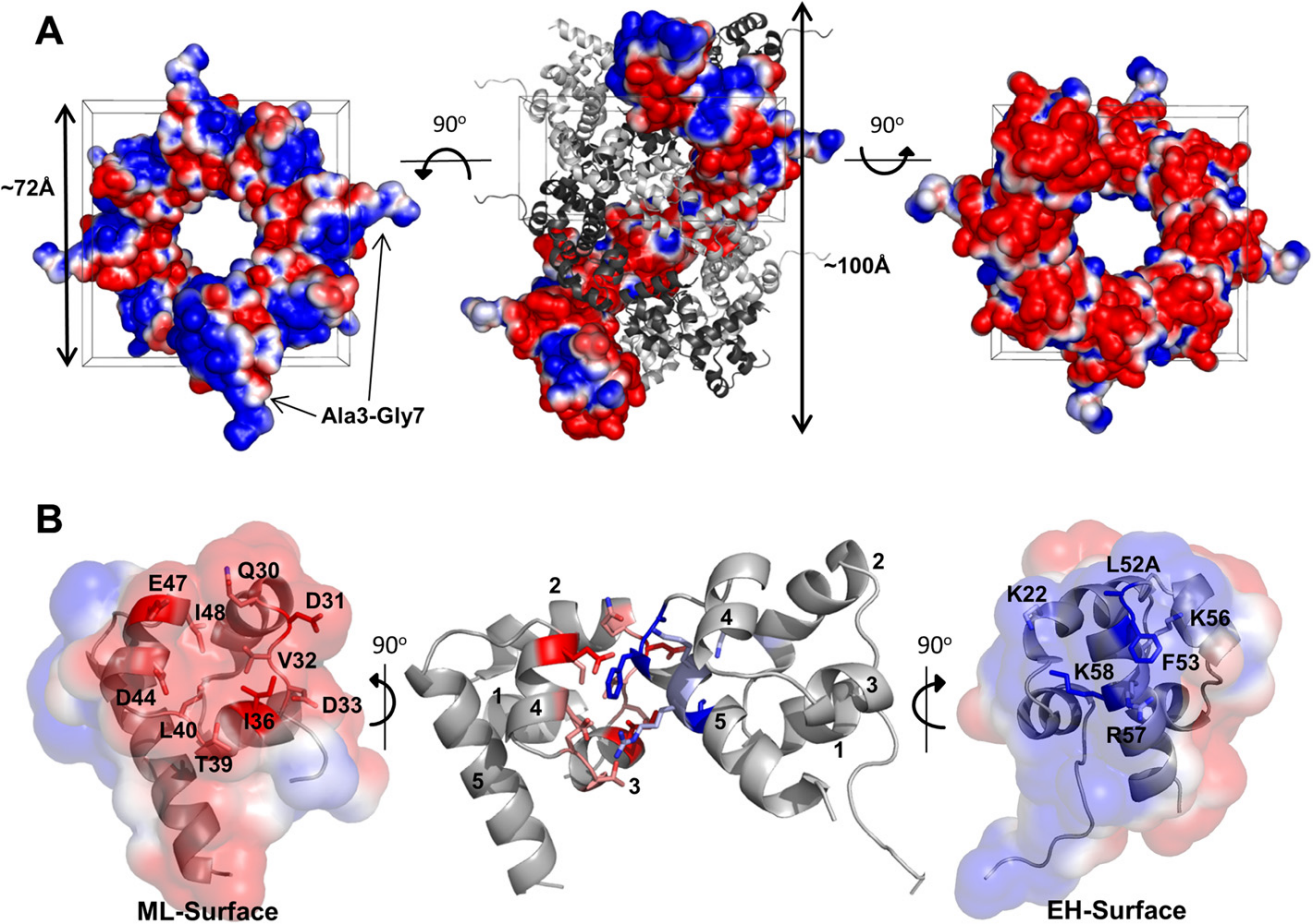
**Structure of the ANKS3-SAM L52A mutant. A)** ANKS3-SAMs pack as a triple helix approximately 72 Å in diameter and 100 Å long per helical repeat. In the side view of the triple helix, two separate polymer chains are shown as cartoons (grey and black) and the third is shown as a space-filled model colored by surface electrostatics calculated using APBS in Pymol and contoured at ±1 kT/e; red is negatively charged and blue is positively charged. Looking down the polymer axis (left and right) and viewing only the surface electrostatics of a single polymer for clarity reveals the charge complementarity of the polymer surface. **B)** A single ANKS3-SAM homodimer is shown. Helices have been numbered 1–5. Side chains of residues found critical for the EH-and ML-surface in the negGFP binding assay are shown colored in blue and red, respectively. Both the EH- and ML-surfaces are shown colored by surface electrostatics calculated using APBS and contoured at ±1 kT/e, revealing the charge complementarity of the binding interface.

We were able to obtain a crystal structure of the L52A mutant (Figure 5). ANKS3-SAM L52A crystallized in space group P4_1_ with two molecules in the asymmetric unit and the structure was solved to 1.6 Å resolution. Each SAM domain has the characteristic five α-helical fold and chains in the asymmetric unit are nearly identical (RMSD of 0.29 Å on backbone atoms and 0.3 Å on all atoms). Examination of the crystal packing reveals a triple helix of intertwined SAM polymers (Figure 5A). Individual SAM polymers contain 8 SAMs per helical repeat, with each helical repeat measuring 72 Å in diameter and 100 Å in length. As expected from the mutational studies described above, the ANKS3 SAM domains associate via sequential interactions of ML and EH surfaces (Figure 5B). The ML-surface, formed by residues spanning loop 2 through helix 4, is composed of a shallow hydrophobic patch (residues V32, I36, L40, and I48) flanked by negatively charged residues (D31, D33, D44, E47). The EH-surface encompasses the N-terminal portion of helix 5 and contains a critical Phe (F53) which packs against the hydrophobic patch of a neighboring ML-surface. Several positively charged residues (K22, K56, R57, K58) surround F53, creating a ring of positive charge which binds the ring of negative charge on a neighboring ML-surface through the formation of salt bridges (K22 + D31, K56 + E47, R57 + D44). The striking asymmetric charge distribution is consistent with the strong salt dependence of subunit association (see above).

The crystal structure is consistent with the identified polymer-blocking mutants (Figures 4A and 5B). F53E removes a key hydrophobic contact of the EH-surface and I36E introduces steric overlap and disruption of the ML-surface. The L52A mutation used in the crystal structure is found at the end of helix 5 and appears to remove van der Waals packing and complementary hydrophobic surface at the interface. The mutations D31K and E47K remove ionic interactions. Finally, K58 was also found crucial for polymerization and although it does not form a direct salt bridge in the crystal structure, this residue is near the interface and helps to maintain charge complementarity. It is also possible that the interface is altered somewhat in the mutant structure, weakening and/or breaking some salt bridges. The width of the single polymer (~7 nm) is thicker than the polymers seen by TEM (4–6 nm) but SAM domain polymers are known to stretch and compact readily in different crystal forms [40].

Individual ANKS3-SAM polymers pack in the crystal structure as a striking triple helix, but we do not know if this is a biologically relevant structure. Caskin1, which also contained 8 SAMs per helical polymer repeat, adopted a triple helix in the crystal, but it was shown to likely be an artifact of crystallization [20]. The N- and C-termini do splay out from the triple helix, so the remaining domains of the full-length protein could be accommodated. Moreover, there is charge complementarity between polymer surfaces. In particular, the polymer surface viewed from the free ML-surface end (Figure 5A right) has negative patches which complement the positive patches seen from the free EH-surface end (Figure 5A left). Unlike the inter-polymer association seen in the Caskin1 structure which was largely mediated by an added His_6_-tag, the triple helix of ANKS3-SAM is held together by residues Ala3-Gly7 of chain A (for which there is no equivalent density in chain B) intercalating between SAM polymers of an adjacent triple helix (Additional file 2). Nevertheless, the polymers are not packed tightly, as there are ample gaps between individual polymers.

The phenomenon of ANKS3-SAM polymer sheet formation observed by TEM also remains to be tested in the context of the full-length protein. Sheets of triple helices can be constructed from the crystal structure, but the sheets formed in the crystal packing (Additional file 2) would be incompatible with triple helix formation. Although the N- and C- termini extend away from the triple helix polymer axis, additional domains of the full-length protein cannot be obviously accommodated in the sheets. If sheets do form as seen in the EM images, it would require different packing than we see in the crystal structure.

### The EH-surface of ANKS3-SAM binds the ML-surface of ANKS6-SAM

To map the binding interface between ANKS3-SAM and ANKS6-SAM, we employed the negGFP binding assay discussed above. As shown in Figure 4B, mutations in residues L52, F53, and K58 on the EH-surface of ANKS3-SAM and mutations in residues E29 and D42 on the ML-surface of ANKS6-SAM abolish the ANKS3-SAM + ANKS6-SAM hetero-interaction in this assay.Since the EH-surface of ANKS3-SAM is required for both polymerization and binding to ANKS6-SAM, these two events are mutually exclusive. Therefore, ANKS6-SAM binding to ANKS3-SAM should block ANKS3-SAM polymerization. To test this possibility, we mixed ANKS6-SAM with the insoluble ANKS3-SAM polymer described above. As shown in Figure 4C, the addition of ANKS6-SAM does indeed lead to solubilization of the ANKS3-SAM precipitate.

The ability of ANKS6-SAM to solubilize ANKS3-SAM polymers and the slower migration of the hetero-interaction on the native gel compared to ANKS3-SAM suggests that ANKS6-SAM has a higher affinity for ANKS3-SAM than ANKS3-SAM does for itself. To determine the affinity of the ANKS3-SAM/ANKS6-SAM interface we again used SPR by immobilizing ANKS6-SAM and measuring equilibrium binding to an ANKS3-SAM ML-surface mutant, I36E. At 0.15 M NaCl, we observed a high binding affinity (K_d_ = 249 ± 8 nM) (Figure 4E). This affinity is more than an order of magnitude tighter than the binding affinity we measured for the native EH-ML interface formed between ANKS3-SAMs (K_d_ = 5.8 ± 0.4 μM), indicating that ANKS6-SAM binding could effectively compete with polymerization. Binding is also strongly salt dependent as shown in Figure 4F, suggesting that ionic interactions are important features of the interface.

### Structure of the ANKS3-SAM/ANKS6-SAM complex

**Figure 6:**
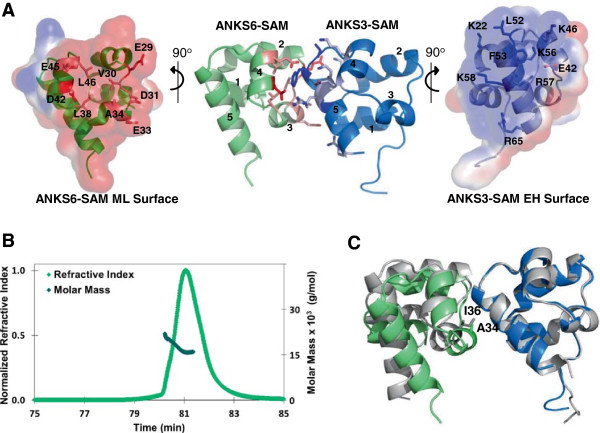
**Structure of the ANKS3-SAM/ANKS6-SAM heterodimer. A)** The ANKS3-SAM EH-surface binds the ANKS6-SAM ML-surface. Helices of the individual SAM domains are numbered 1–5. Side chains of residues which form the ML- and EH-surface are shown and colored red and blue, respectively. Each surface is also colored by surface electrostatics calculated using APBS and contoured at ±1 kT/e, revealing the charge complementarity of the binding interface. **B)** SEC-MALS analysis of a 1:1 molar ratio mix of ANKS3-SAM I36E + ANKS6-SAM wt produces a single monodisperse peak with a calculated molecular weight of 16.8 kDa, which corresponds to a homogenous population of heterodimer. **C)** Alignment of an ANKS3-SAM homodimer (grey) with the ANKS3-SAM/ANKS6-SAM heterodimer (blue-green), formed by aligning the backbone atoms of the common ANKS3-SAMs. I36 in ANKS3-SAM is changed to A34 in ANKS6-SAM, which allows ANKS6-SAM to tilt closer to ANKS3-SAM and form more interactions.

To learn how ANKS3-SAM and ANKS6-SAM bind each other, we determined a crystal structure of the complex (Figure 6A). To prevent ANKS3-SAM polymerization without destroying ANKS6-SAM binding, we mixed an ML-surface mutant of ANKS3-SAM, I36E, with wild-type ANKS6-SAM. These proteins formed a heterodimer when analyzed by SEC-MALS (Figure 6B) and yielded crystals suitable for structure determination.

The ANKS3-SAM/ANKS6-SAM heterodimer crystallized in space group C222_1_ with four molecules (2 ANKS3-SAM I36E mutants and 2 ANKS6-SAMs) per asymmetric unit. Both of the ANKS3-SAM chains and both ANKS6-SAM chains in the asymmetric unit had closely similar structures, with RMSDs between backbone atoms of 0.279 Å and 0.169 Å, respectively. Additionally, the overall structure of ANKS3-SAM I36E aligns well with ANKS6-SAM (RMSD across backbone atoms is 0.626 Å) with the only obvious difference being a 1.6 Å outward shift of ANKS6-SAM’s helix 3.Similar to the ML-surface of ANKS3-SAM, the ML-surface of ANKS6-SAM contains a shallow hydrophobic patch (residues V30, A34, L38, L46) flanked by negatively charged residues (E29, D31, E33, D42, E45) (Figure 6A). Many of these charged residues form salt bridges with ANKS3-SAM (ANKS6 E29 + ANKS3 K22, ANKS6 D31 + ANKS3 K58, ANKS6 D42 + ANKS3 R57). Other residues of ANKS6-SAM (E33, E45) form hydrogen bonds with ANKS3-SAM bridged by water. These results are consistent with our native gel analysis of critical interface residues (Figure 4B). Similar to the ANKS3-SAM/ANKS3-SAM interface, the F53E mutation in ANKS3-SAM removes a Phe required for packing against the hydrophobic patch of the ANKS6-SAM ML-surface and the L52E mutation removes van der Waals interactions and hydrophobic contacts that otherwise stabilize the interaction interface. Removal of salt bridges by the mutations K58E in ANKS3-SAM, E29K in ANKS6-SAM, and D42K in ANKS6-SAM also break the interface. The ANKS6-SAM mutation R54W (R823W in the full length protein) breaks the interaction with ANKS3-SAM as well and will be discussed below.

Comparing an ANKS3-SAM/ANKS6-SAM heterodimer with an ANKS3-SAM homodimer by aligning the ANKS3-SAMs, we see that ANKS6-SAM is tilted closer towards ANKS3-SAM (Figure 6C). This ANKS3-SAM/ANKS6-SAM interface buries on average 454 Å^2^ of surface area, which is approximately 100 Å^2^ more than was buried at the same interface in the ANKS3-SAM polymer. An isoleucine (I36) in ANKS3-SAM is switched for an alanine (A34) in ANKS6-SAM at the ML-surface. The decreased steric bulk of Ala compared to Ile allows helix 3 of ANKS6-SAM’s ML-surface to approach the EH-surface of ANKS3-SAM more closely, resulting in the burial of additional surface area and the formation of more salt bridges and hydrogen bonds, which may explain the higher affinity of the ANKS3-SAM/ANKS6-SAM interface. The ANKS6-SAM structure also shows that the EH-surface is lacking a large hydrophobic residue flanked by positively charged residues, thereby showing that ANKS6-SAM cannot polymerize because its EH-surface is incompatible with binding its ML-surface.

### The R823W mutation of Pkd/Mhm(cy/+) rats perturbs the structure of ANKS6-SAM

**Figure 7:**
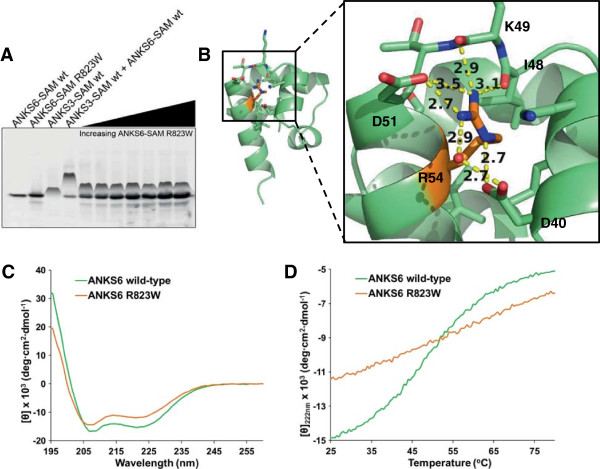
**Characterizing the Cy mutation. A)** negGFP native gel assay of ANKS3-SAM, ANKS6-SAM wt and ANKS6-SAM R823W. A titration series with a constant amount of ANKS3-SAM and increasing amount of ANKS6-SAM R823W is unable to restore the ANKS3-SAM/ANKS6-SAM interaction. **B)** The Cy mutation (R54) according to our numbering is highlighted on the ANKS6-SAM crystal structure in orange. R54 forms salt bridges and hydrogen bonds with nearby atoms to stabilize the fold in this part of the domain. **C)** CD spectra of ANKS6-SAM wt and ANKS6-SAM R823W. The reduced CD signal at 222 nm and the shifted minima around 208 nm correlate with a loss of alpha-helical character and gain of random coil. **D)** Thermal denaturation curves of ANKS6-SAM wt and ANKS6-SAM R823W monitored by CD signal at 222 nm. ANKS6-SAM wt exhibits a broad, weakly cooperative unfolding curve with T_m_ approx. 48°C, while ANKS6-SAM R823W unfolding is completely uncooperative.

Using our native gel binding assay, we discovered that the Cy mutation (R54W in our numbering of the construct, R823W in the full-length protein) in ANKS6-SAM destroys the interaction with ANKS3-SAM (Figure 4B). This interaction could not be restored by titrating increasing amounts of ANKS6-SAM R823W with ANKS3-SAM (Figure 7A). To understand how the Cy mutation might be responsible for disease in Pkd/Mhm(cy/+) rats, we examined its position in our structure of ANKS6-SAM. R823 (R54 in the crystal structure numbering, see Figure 1) forms salt bridges with D51 of helix 5 and D40 of helix 4, and forms hydrogen bonds with the backbone carbonyls of I48 and K49 which both lie on a loop between helices 4 and 5 (Figure 7B). Through these interactions, R54 is involved in stapling helices 4 and 5 and in maintaining the overall fold in this segment of the domain.

Since the R823W mutation does not lie on either the ML- or EH-surface of ANKS6-SAM, we reasoned that its ability to abolish binding to ANKS3-SAM must be due to a long range structural alteration. Indeed the far-UV CD spectrum of ANKS6-SAM R823W shows an approximately 10% loss of helical structure with a parallel increase in random coil compared to the wild-type protein (Figure 7C). Moreover, the stability of the protein is dramatically reduced. As shown in Figure 7D, wild-type ANKS6-SAM displayed a broad, reversible unfolding curve with a T_m_ of approximately 48°C. In contrast, the ANKS6-SAM R823W mutant strikingly exhibited a complete loss of cooperative unfolding. Therefore, the R823W mutation appears to dramatically destabilize the structure. The mutant protein also migrates slightly faster and with a higher molecular weight as assessed by SEC-MALS (Additional file 3), consistent with generalized unfolding. Indeed, modeling a tryptophan at position R54 in our structure is impossible as this would introduce severe steric clashes. Thus, coupling the removal of Arg with the insertion of a Trp disturbs the overall tertiary structure of the protein and prevents the ML-surface of the ANKS6-SAM domain from adopting a fold complementary to binding the ANKS3-SAM domain.

## Discussion

The PKD/Mhm(cy/+) rat, in which an R823W mutation in the SAM domain of ANKS6 is causal of disease, has been used extensively as an animal model for the study of human ADPKD. However, the underlying mechanism whereby this single mutation leads to improper kidney development and loss of renal function has been unclear. For the first time, we have identified the SAM domain of ANKS3 as a direct binding partner of the ANKS6-SAM domain and shown that this interaction is lost in the ANKS6-SAM R823W mutant. The physiological relevance of this interaction is supported by the independent identification of the potential ANKS3/ANKS6 interaction in a proteomics screen [13].

By solving the crystal structure of ANKS3-SAM, we have observed that ANKS3-SAM forms polymers via sequential interactions of ML- and EH-surfaces, much like other SAM domains. The polymer formation by ANKS3-SAM suggests that ANKS3 may be capable of scaffolding a larger protein complex. We also observed higher order structure of the polymer, including a triple helix in the crystal and sheet structures in TEM images, but the biological relevance of these polymer associations is unclear. SAM domains have been previously found to organize as sheets of polymers that require divalent metal cation binding to form [35,36], but we did not observe any obvious metal binding sites in the ANKS3-SAM crystal structure.

By solving a crystal structure of the ANKS3-SAM/ANKS6-SAM heterodimer, we observed that the EH-surface of ANKS3-SAM binds the ML-surface of ANKS6-SAM. We have also provided a molecular explanation for the defect in the R823W mutation: it disrupts the tertiary structure of the ANKS6-SAM domain and prevents the ML-surface of ANKS6-SAM from adopting a conformation capable of binding ANKS3-SAM. Because the EH-surface of ANKS3-SAM is mutually exclusive for binding either the ML-surface of ANKS3-SAM or the ML-surface of ANKS6-SAM, we expect that ANKS6 may act as a polymer capper [24,28]. It may shorten the ANKS3-SAM polymers or it may simply recruit ANKS3 polymers to a larger complex of proteins.

ANKS6 has been linked to Bicaudal-C1 (BICC1), a protein that when mutated is responsible for disease in the *jcpk* mouse model of ADPKD and the *bpk* mouse model of ARPKD (autosomal recessive polycystic kidney disease) [41,42]. ANKS6 and BICC1 have been shown to co-immunoprecipitate and co-localize in inner medullary collecting duct (IMCD) cells. An unidentified protein complex has been suggested to link ANKS6 to BICC1, by binding both the ANKS6-SAM domain and a strand of RNA to which the KH domains of BICC1 are bound [42]. It is reasonable to suspect that ANKS3 may also be part of a complex with ANKS6 and BICC1.

Transgenic overexpression of ANKS6^(p.R823W)^ can generate a disease phenotype, indicating that ANKS6-SAM R823W acts as a dominant negative [12]. A dominant negative effect would be expected to arise from a gain of function, yet the mutation clearly destabilizes the structure of the SAM domain. A reasonable possibility is that other domains on ANKS6 recruit it to a complex [42], but the defective SAM domain fails to appropriately recruit other proteins, such as ANKS3, leading to defective function. Altered RXR-mediated signaling pathways are observed in PKD/Mhm(cy/+) rats [43] and ANKS6 has recently been placed as a central node in a distinct NPHP-associated signaling network [13].

The recent finding of mutations in ANKS6 in individuals presenting with polycystic kidney disease [13] has catapulted this protein from relevance in an animal model to having direct implications for human renal development and function. Furthermore, the fact that one patient with a truncation in ANKS6 at the N-terminal end of the SAM domain also presented with aortic stenosis, causing obstructive cardiomyopathy, implicates ANKS6 in cardiac development and function [13]. Whether ANKS3 may also have a role in cardiac development, via its link with ANKS6, remains to be investigated. So far, ANKS3 is the only direct binding partner of ANKS6 identified that is affected by the R823W mutation. Our work provides a structural and biochemical basis for future work on ANKS3 and its interaction with ANKS6.

## Conclusions

We have identified and characterized the novel interaction between the SAM domain of ANKS3 and the SAM domain of ANKS6. ANKS3-SAM was found to be capable of polymerization, although polymer formation and binding to ANKS6 appear mutually exclusive, suggesting ANKS6 may act as a polymer capper. The R823W mutation associated with cystic kidney disease causes a destabilization of the ANKS6 SAM domain which disrupts binding to ANKS3. By structurally and biochemically characterizing this new interaction we provide a foundation to support continued research into how the ANKS3/ANKS6 interaction affects kidney function.

## Methods

### Cloning and mutagenesis

negGFP-human-SAM fusions and DNA used in cloning were as described previously [22]. All human ANKS3-SAM constructs contained residues 421–490 [UniProt:Q6ZW76] and all human ANKS6-SAM constructs contained residues 771–840 [UniProt:Q68DC2]. A His_6_-tagged construct of ANKS3-SAM was generated by cloning the human ANKS3-SAM sequence into a pET28 vector (Novagen). A His_6_-tagged construct of ANKS6-SAM was generated by cloning the ANKS6-SAM sequence into a pBAD-HisA vector (Invitrogen). In both constructs, the residues MARHHHHHHSSG were added to the N-terminus of each SAM to incorporate a His_6_-tag. Hexahistidine small ubiquitin-like modifier (SUMO) tagged constructs were generated by cloning the ANKS3-SAM and ANKS6-SAM sequences into a pHis-SUMO vector [44]. Site-directed mutagenesis was performed using the Quickchange method (Agilent). All plasmid sequences were verified by DNA sequencing (Genewiz).

### negGFP native gel binding assay

negGFP-human-SAM fusions transformed into ARI814 cells [45] were expressed as described previously [22]. Harvested cells were resuspended in 0.5 mL of 20 mM Tris pH 7.5, 0.3 M NaCl, 2 mM TCEP, supplemented with DNaseI (40 μg/mL), lysozyme (10 mg/mL), PMSF (1 mM), MgCl_2_ (10 mM), and half a tablet of cOmplete mini, EDTA-free protease inhibitor (Roche) and lysed by three freeze-thaw cycles and one 10-sec. round of sonication. Lysate was centrifuged at 16,060 *g* for 20 minutes at 4°C and the pellet discarded. The expression levels of the negGFP-human-SAM fusions was determined by fluorescence intensity as described previously [22]. To identify new ANKS3-SAM interactions, lysates were mixed to maintain equal amounts of negGFP-SAM fusion (dilutions were made using 20 mM Tris pH 7.5, 0.3 M NaCl, 1 mM TCEP). Mixes were allowed to equilibrate at 4°C for 3 hours, at which point 7.5 μL of 4X RunBlue Native Sample Buffer (Expedeon) was added and samples were applied to a 20% RunBlue 12-well Native gel in RunBlue Native Run Buffer (Expedeon) and developed at 90 V for 24 hours at 4°C. Gels were visualized on a Bio-Rad Molecular Imager FX Pro-Plus using an excitation wavelength of 488 nm and an emission wavelength of 510 nm. For characterization of ANKS3-SAM and ANKS6-SAM mutants, lysates were prepared as above and adjusted by fluorescence for equal gel loading. Lysates were mixed in varying ratios based on fluorescence where appropriate, and allowed to equilibrate at 4°C for 4 hours before gel loading. Gels were run at 90 V for 15 hours at 4°C and visualized as above.

### Protein expression and purification

#### negGFP-ANKS3-SAM fusion

negGFP-ANKS3-SAM fusion transformed into ARI814 cells [45] was expressed as described previously [22]. Harvested cells were lysed as described previously, except that 5 cOmplete mini, EDTA-free protease inhibitor tablets (Roche) were included in the lysis buffer and five 1-min. rounds of sonication were used [22]. Following centrifugation at 13,200 *g* for 20 minutes, lysate supernatant was supplemented with 10 mM imidazole and rocked with 2 mL of Ni-NTA Superflow agarose (Qiagen) for 1 hour at 4°C. The resin was washed with lysis buffer (20 mM Tris pH 7.5, 1 M NaCl, 1 mM TCEP) containing 20 mM imidazole, and eluted with lysis buffer containing 75 mM imidazole. Eluted protein was dialyzed into 20 mM Tris pH 7.5, 0.15 M NaCl, 1 mM TCEP and loaded onto a 5 mL HiTrap Q HP column (GE) 4°C. Protein was eluted using a shallow gradient of NaCl (0.25-0.5 M) in 20 mM Tris pH 7.5, 1 mM TCEP. Purified protein was dialyzed into 20 mM Tris pH 7.5, 0.3 M NaCl, 1 mM TCEP and concentrated using an Amicon Ultra (10 kDa MWC0) centrifugal filter unit (Millipore) to a final concentration of 4.3 mg/mL.

#### His_6_-tagged constructs

His_6_-tagged ANKS6-SAM was transformed into ARI814 cells and expressed as above in 6 L of LB media supplemented with 100 μg/mL ampicillin. Harvested cells were resuspended to 80 mL in lysis buffer (20 mM Tris pH 8, 0.5 M NaCl) containing PMSF (1 mM), DNaseI (20 μg/mL), and MgCl_2_ (10 mM) and lysed on an EmulsiFlex-C3 (Avestin) at 18,000 psi. Lysate was centrifuged at 13,200 *g* for 20 minutes at 4°C. Supernatant, with imidazole added to 10 mM, was bound at 4°C to a 5 mL HiTrap IMAC HP column (GE) charged with NiCl_2_. The column was washed with lysis buffer containing 20 mM imidazole and eluted using a shallow gradient of imidazole (20-260 mM). Eluted protein was diluted to approximately 1 mg/mL and dialyzed into 20 mM Tris pH 8.0, 50 mM NaCl. Protein was further purified by loading onto a 1 mL HiTrap Q column (GE) equilibrated in 20 mM Tris pH 8 at 4°C. Protein was eluted using a shallow gradient of NaCl (0–1.0 M) in 20 mM Tris pH 8.0. Fractions containing pure protein were dialyzed against 20 mM Tris pH 8.0, 0.3 M NaCl.

His_6_-tagged ANKS3-SAM was transformed into Rosetta(DE3) cells (Novagen) and 4.5 L of culture was grown in LB media supplemented with 30 μg/mL kanamycin and 34 μg/mL chloramphenicol. Cells were grown at 37°C with shaking until cell density reached an OD_600_ of 0.7, at which point cells were chilled to 18°C, induced with 1 mM isopropyl β-ᴅ-galactopyranoside (IPTG), and grown an additional 16 hours at 18°C. Harvested cells were resuspended to 80 mL in lysis buffer (20 mM Tris pH 7.0, 1 M NaCl, 2 mM BME) containing PMSF (1 mM), DNaseI (20 μg/mL), MgCl_2_ (5 mM) and processed as above. Supernatant, with imidazole added to 10 mM, was rocked with 3 mL of Ni-NTA Superflow agarose (Qiagen) for 1 hour at 4°C. Resin was washed with 50 mL of lysis buffer containing 10 mM imidazole, 200 mL of lysis buffer containing 20 mM imidazole, and eluted using lysis buffer with 200 mM imidazole. Elution fractions were stored at 4°C and developed fluffy precipitate overnight and for several days thereafter.

#### SUMO-fusions

pHis-SUMO constructs (ANKS3-SAM mutants L52A, F53E, I36E, and ANKS6-SAM wt and R823W) were transformed into Rosetta(DE3) pLysS cells (Novagen) and 4 L of culture was grown and expressed as described above. Harvested cells were resuspended in lysis buffer (50 mM NaHPO_4_ pH 8.0, 0.5 M NaCl, 2 mM BME) containing PMSF (1 mM), DNaseI (20 μg/mL), and MgCl_2_ (5 mM) and lysed on an EmulsiFlex-C3 (Avestin) at 18,000 psi followed by centrifugation at 13,200 *g* for 20 minutes at 4°C. Supernatant, with imidazole added to 10 mM, was rocked with Ni-NTA Superflow agarose (Qiagen) for 1 hour at 4°C. Resin was washed with lysis buffer containing 20 mM imidazole and eluted with lysis buffer containing 250 mM imidazole. The different proteins were dialyzed into buffers of various ionic strengths as required to maintain solubility: (1) ANKS3-SAM F53E was dialyzed into 20 mM Tris pH 8.0, 0.3 M NaCl, 2 mM BME; (2) ANKS6-SAM wt and R823W were diluted to 5-7 mg/mL and dialyzed into 20 mM Tris pH 8.0, 0.3 M NaCl; (3) ANKS3-SAM L52A and I36E were diluted to 4-5 mg/mL and 50 mM EDTA was added to prevent precipitation seemingly induced by leached Ni^2+^, followed by extensive dialysis into 20 mM Tris pH 8.0, 0.5 M NaCl, 2 mM BME. To remove the His_6_-SUMO tag, all constructs were digested with the His_6_-tagged catalytic domain of SUMO protease 1 (ULP1) at a 50:1 protein:protease molar ratio for 16 hours at 4°C [46]. The cleaved His_6_-SUMO tag and ULP1 protease were removed by two rounds of subtractive Ni-NTA. Here the protein was either in the flow-through (ANKS3-SAM F53E, ANKS6-SAM wt and R823W) or remained weakly bound to the resin and was eluted with 20 mM NaHPO_4_ pH 8.0, 0.5 M NaCl, 2 mM BME, 10 mM imidazole (ANKS3-SAM L52A and I36E). Final purification steps are specific to each construct as described below.

#### ANKS3-SAM F53E

ANKS3-SAM F53E from the subtractive Ni-NTA flow-through was dialyzed into 20 mM Tris pH 7.5, 30 mM NaCl, 1 mM DTT and bound to a 5 mL HiTrap Q HP (GE) column equilibrated in 20 mM Tris pH 7.5, 2 mM DTT. Protein was eluted using a shallow gradient of NaCl (0.1-0.5 M). Fractions containing pure ANKS3-SAM F53E were dialyzed into 20 mM Tris pH 8.0, 50 mM NaCl, 2 mM BME and concentrated in an Amicon Ultra (3 kDa MWCO) centrifugal filter unit (Millipore) to 27 mg/mL.

#### ANKS6-SAM wt and ANKS6-SAM R823W

ANKS6-SAM wt and R823W from the subtractive Ni-NTA flow-through were each dialyzed into 20 mM Tris pH 8.0, 50 mM NaCl and bound to a 5 mL HiTrap Q HP column (GE) equilibrated in 20 mM Tris pH 8.0. Protein was eluted with a shallow gradient of NaCl (0.1-1 M). Pure fractions were pooled and dialyzed against 20 mM Tris pH 8, 0.15 M NaCl. Proteins were concentrated in Amicon Ultra (3 kDa MWCO) centrifugal filter units to 12–14 mg/mL.

#### ANKS3-SAM L52A

ANKS3-SAM L52A exhibited a weak affinity for IMAC resin and was dialyzed into 20 mM Tris pH 8.0, 0.5 M NaCl, 2 mM BME and bound to a 5 mL HiTrap IMAC HP column (GE) charged with NiSO_4_. Protein was eluted using a shallow gradient of imidazole (0-15 mM). Fractions containing pure ANKS3-SAM L52A were dialyzed into 20 mM Tris pH 8.5, 0.15 M NaCl, 1 mM DTT and concentrated using an Amicon Ultra (3 kDa MWCO) centrifugal filter unit (Millipore) to approx. 5 mg/mL. Surprisingly, at this concentration the protein spontaneously formed crystalline needles with low resolution diffraction. Needles were resolubilized by the addition of NaCl to a final concentration of 0.72 M.

#### ANKS3-SAM I36E

ANKS3-SAM I36E eluted from the subtractive Ni-NTA column in both the flow-through and the 10 mM imidazole wash. The protein was dialyzed into 20 mM Tris pH 8.0, 0.5 M NaCl, 2 mM BME and bound to a 5 mL HiTrap IMAC HP column (GE) charged with NiSO_4_. Protein was eluted using a shallow gradient of imidazole (0-15 mM). Pure fractions were pooled and dialyzed into 20 mM Tris pH 8.0, 0.75 M NaCl, 1 mM DTT. Protein was concentrated in an Amicon Ultra (3 kDa MWCO) centrifugal filter unit (Millipore) to approx. 2.5 mg/mL.

### Resolubilization of ANKS3-SAM precipitate by ANKS6-SAM

His_6_-tagged ANKS3-SAM precipitate was formed by dialyzing protein at 3.2 mg/mL into low salt buffer (20 mM Tris pH 7, 0.25 M NaCl, 1 mM DTT). His_6_-tagged ANKS6-SAM at 3.3 mg/mL in 20 mM Tris pH 8.0, 0.3 M NaCl buffer or buffer alone (20 mM Tris pH 8.0, 0.3 M NaCl) was added in varying ratios of 2:1, 1:1, and 1:2 (ANKS3-SAM vs. ANKS6-SAM ratio) to the ANKS3-SAM precipitate, keeping the total volume and total concentration of protein constant. Mixes were allowed to incubate on ice for 3 hours.

### Crystallization and structure determination

#### ANKS3-SAM L52A

Initial screening for crystallization conditions was performed at the High Throughput Macromolecular Crystallization Facility at UCLA. The crystallization trials were carried out in hanging drops by vapor diffusion, using commercially available screens and a Mosquito TTP crystallization Robot.

Native crystals were grown by hanging drop vapor diffusion by mixing 1μL of ANKS3-SAM L52A at 4.3 mg/mL with 2μL of well solution (0.1 M Na/KPO_4_ pH 6.6, 0.3 M NaCl, 15% PEG-8000). Rod-shaped crystals grew at 4°C over a two-week period and were cryoprotected using well solution supplemented with 25% glycerol. For phasing, crystals were briefly soaked and cryoprotected (5 sec) in a solution of 0.5 M KI prepared in well solution supplemented with 27% glycerol. A data set was collected for a single ANKS3-SAM L52A iodide derivative crystal cryo-cooled to 100 K at UCLA using the in-house RIGAKU FRE + generator and HTC image plate detector at a wavelength of 1.5418 nm. Data reduction and scaling were performed using XDS/XSCALE [47]. Phasing was accomplished by single anomalous dispersion (SAD) using the HKL2MAP interface and SHELX program [48,49]. Ten iodide atoms were detected and used for phasing. Density modification and model building were accomplished using DM and BUCCANEER, respectively, in the CCP4 suite [50]. The structure was briefly refined in PHENIX [51] with inspection and model rebuilding in COOT [52].

A high resolution data set was collected on a single native crystal cryo-cooled to 100 K at the Advanced Photon Source (Argonne National Laboratory), APS-NECAT beamline 24-ID-C on a DECTRIS-PILATUS 6 M detector. Single crystals were mounted with CrystalCap HT Cryoloops (Hampton Research, Aliso Viejo). A data set containing 418 1.0° oscillation frames was collected from a single large crystal at a wavelength of 0.9795 Å. This dataset was indexed and merged for scaling using XDS [47]. The model was refined using PHENIX with TLS parameterization including individual sites and individual atomic displacement parameters [51]. Data collection and refinement statistics are reported in Table 1. The coordinates have been deposited in the PDB with accession code 4NJ8.

**Table 1:** Crystallographic data collection and refinement statistics

	**Iodide derivative of ANKS3-SAM L52A**	**ANKS3-SAM L52A**	**ANKS3-SAM/ANKS6-SAM heterodimer**
**PDB Accession #**		4NJ8	4NL9
**Data collection**			
Location	UCLA	APS 24-ID-C	APS 24-ID-C
Space group	P4_1_	P4_1_	C222_1_
Cell dimensions			
*a*, *b*, *c* (Å)	71.62, 71.62, 33.40	71.89, 71.89, 33.54	47.70, 108.52, 101.74
*α, β, γ* (°)	90.0, 90.0, 90.0	90.0, 90.0, 90.0	90.0, 90.0, 90.0
Resolution (Å)	2.34	1.60	1.50
*R*_sym_	0.118 (.850)	0.087 (.804)	0.069 (.417)
*I*/σ*I*	13.70 (2.62)	20.11 (5.40)	14.83 (4.16)
CC_1/2_	99.7 (74.0)	99.7 (91.7)	99.8 (89.9)
Completeness (%)	99.6 (95.2)	99.3 (99.3)	98.7 (96.9)
Redundancy	6.61 (5.84)	15.15 (15.01)	5.32 (4.94)
**Phasing statistics**			
Number of sites	10		
Mean figure of merit			
SAD/after density modification	0.642/0.813		
MapCC (SHELXE)	0.844		
CC (%)	66.73		
**Refinement**			
Resolution (Å)	2.34	1.60	1.50
No. reflections	7333	22,797	42,203
*R*_work/_*R*_free_	0.2709/0.3478	0.1975/0.2177	0.1798/0.2048
No. atoms			
Protein	1030	1022	1952
Water	-	50	246
Magnesium	-	-	1
B-factors (Å^2^)			
Protein	36.70	37.42	21.92
Water	-	38.73	28.94
R.m.s deviations			
Bond lengths (Å)	0.011	0.006	0.005
Bond angles (º)	1.303	0.920	0.944

Highest resolution shell is shown in parenthesis.

*R*_*sym*_ = ∑ |I − < I > |/∑ < I >, where I is the observed intensity and <I> is the average intensity from observations of symmetry-related reflections. CC_1/2_ = correlation coefficient between two halves of the data [53]. *R*_*work*_ = ∑ |F_obs_ – F_calc_|/∑F_obs_, where F_obs_ and F_calc_ are the observed and calculated structure factor amplitudes, respectively. *R*_*free*_ is calculated for a set of reflections (10%) that were not included in atomic refinement.

#### ANKS3-SAM/ANKS6-SAM heterodimer

Crystals were grown in 2μL hanging drops by mixing equal parts ANKS3-SAM/ANKS6-SAM heterodimer at 30 mg/mL (in 20 mM Tris pH 8.0, 0.15 M NaCl, 2 mM BME) with 0.1 M Tris pH 8.0, 0.25 M MgCl_2_, 31.5% PEG-4000 reservoir solution. Drops were allowed to equilibrate overnight and were streak seeded the following day using a cat whisker to transfer nuclei from crystals that spontaneously formed overnight in a condition with a higher percentage of PEG-4000. Seeded crystals grew within one day and reached full size within 4 days. An x-ray diffraction data set containing 300 1° oscillation frames was collected on a single crystal cryo-cooled to 100 K at the Advanced Photon Source (Argonne National Laboratory), APS-NECAT beamline 24-ID-C, at a wavelength of 0.9793 Å and processed using XDS [47]. Molecular replacement was performed using PHASER with ANKS3-L52A as a search model [54] and refined as described above. Data collection and refinement statistics are reported in Table 1. The coordinates have been deposited in the PDB with accession code 4NL9.

### Structure analysis

Final structure models were validated with the following structure validation tools: PROCHECK [55], ERRAT [56], and VERIFY3D [57]. Graphics were prepared using PyMOL [58]. Surface electrostatics were prepared using the Adaptive Poisson-Boltzmann Solver (APBS) [59] plugin in Pymol and all surfaces were contoured at ±1 kT/e. Analysis of protein-protein binding interfaces was done using the PISA server [60].

### Circular dichroism

Spectra were collected for protein samples at 0.2 mg/ml in 10 mM Tris pH 8.0, 75 mM NaCl at 25°C on a JASCO J-715 circular dichroism spectrophotometer equipped with a Peltier temperature control. Spectra were analysed for secondary structure content using the Selcon, Neural Network, and Contin algorithms available in SoftSec (Softwood Inc.). Thermal melts were performed by monitoring the change in CD signal at 222 nm across a temperature range of 25-80°C, with ramping of 1°C per minute.

### Surface plasmon resonance

Experiments were performed at 21°C in 20 mM HEPES pH 7.5, 1 mM DTT, 0.04% IGEPAL CA-630, and NaCl ranging from 0.1-0.3 M using a Biacore T100 (GE). To determine the binding affinity of the ANKS3-SAM/ANKS6-SAM interface, ANKS6-SAM wt was immobilized on a Biacore CM5 chip (GE) via EDC/NHS crosslinking. To determine the binding affinity of the ANKS3-SAM/ANKS3-SAM interface, ANKS3-SAM F53E was immobilized on a Biacore CM5 chip (GE) via EDC/NHS crosslinking. In both cases, ANKS3-SAM I36E at varying concentrations was passed over the chip and equilibrium binding levels were measured. All data points were taken in triplicate and binding data was fit to a 1:1 steady-state model using Biacore T100 Evaluation software. In total, 630.9 response units (RUs) were immobilized on the ANKS6-SAM wt chip surface and 2188.7 RUs were immobilized on the ANKS3-SAM F53E chip surface. At 0.15 M NaCl, calculated R_max_ values were 262.7 for the ANKS6-SAM wt chip and 682.3 for the ANKS3-SAM F53E chip, indicating that 30-40% of the surface molecules were active. To determine how ionic strength impacted binding affinity, the ANKS3-SAM/ANKS6-SAM K_d_ was determined at 0.15, 0.2, 0.25, and 0.3 M NaCl and the ANKS3-SAM/ANKS3-SAM K_d_ was determined at 0.1, 0.15, 0.2, and 0.25 M NaCl. Prior to the experiments, ANKS3-SAM I36E at 2.4 mg/mL was dialyzed into 20 mM HEPES pH 7.5, 0.75 M NaCl, 1 mM DTT, with the high salt required to maintain stability. Immediately prior to each Biacore run, dilutions of ANKS3-SAM I36E were prepared to match the running buffer and protein concentration was determined by Bradford assay, using known concentrations of ANKS3-SAM I36E for a standard curve.

### Electron microscopy

Samples were applied to carbon/formvar coated copper grids (Ted Pella, catalog number 01754-F) made hydrophilic by glow discharge immediately before use and allowed to bind for several minutes. Grids were rinsed with distilled water and negatively stained with 1% uranyl acetate. Samples were analysed on a CM120 transmission electron microscope (TEM) operating at 120 kV. Images were recorded using a TEITZ F224HD CCD camera and processed using ImageJ (NIH).

### SEC-MALS

100 μL of protein (either ANKS3-SAM/ANKS6-SAM heterodimer at 15 mg/mL, ANKS6-SAM wt at 10 mg/mL, or ANKS6-SAM R823W at 10 mg/mL) was analysed by SEC-MALS. Protein was loaded onto a WTC-030S5 analytical size-exclusion column (Wyatt Technology Co.) equilibrated in 20 mM Tris pH 8.0, 0.15 M NaCl, (+2 mM BME for the ANKS3-SAM/ANKS6-SAM heterodimer) using an AKTA purifier (GE) and run at 0.5 mL/min on a miniDAWN TREOS (Wyatt Technology Co.). Eluted protein peaks were analysed for calculated molecular weight and monodispersity using ASTRA software (Wyatt Technology Co.)

## Abbreviations

ADPKD: Autosomal dominant polycystic kidney disease; ANKS3: Ankyrin repeat and SAM-domain containing protein 3; ANKS6: Ankyrin repeat and SAM-domain containing protein 6; Bicc1: Bicaudal C homolog 1; DGKδ: Diacylglycerol kinase delta; EH: End-helix; ML: Mid-loop; negGFP: Green fluorescent protein with a net charge of −30; PKD: Polycystic kidney disease; RXR: Retinoid X receptor; SEC-MALS: Size exclusion chromatography with inline multi-angle light scattering; Shank3: SH3 and multiple ankyrin repeat domains protein 3; SUMO: Small ubiquitin-like modifier; TEM: Transmission electron microscopy.

## Competing interests

The authors declare that they have no competing interests.

## Authors’ contributions

CNL and JUB designed all experiments and CNL performed all the experiments. MJK and CNL developed the negGFP native gel assay. CNL and DC solved the crystal structures of ANKS3 and ANKS6. SH provided valuable consultation on disease implications. CNL and JUB wrote the manuscript. All authors read and approved the final manuscript.

## Supplementary Material

Additional file 1 **Human SAM domains screened for interaction with ANKS3-SAM.** negGFP-SAM-domain fusions of the following human SAM-domain containing proteins were screened for binding to negGFP-ANKS3-SAM using the negGFP native gel assay. Sequences of SAM domains used and cloning are as described previously [22]. Where “2SAMs” is listed, the construct contains two SAM domains in tandem. Click here for file

Additional file 2 **Crystal packing of ANKS3-SAM triple helices. A)** Individual ANKS3-SAM polymers intertwine to create a triple helix. Triple helices pack side-by-side in the crystal structure. A single ANKS3-SAM triple helix fills the unit cell, shown as a boxed outline. Individual polymers in each triple helix are colored blue, grey, and black. A single polymer is shown as a space-filled model colored by surface electrostatics generated using APBS in Pymol and contouring at ± 1kT/e. Within a single ANKS3-SAM polymer, the N-terminal arm (residues Ala3-Gly7) extends outward from every other SAM domain in the helical segment and forms contacts with a neighboring polymer of triple helices. **B)** Closer view of the N-terminal arm swapping that occurs between polymers. Two polymers of triple helices are shown, colored as above. Within each, a single polymer has been removed for clarity. Residues Ala3-Gly7 intercalate between two polymers of an adjacent triple helix, thereby forming crystal contacts which stabilize the observed triple helix. Click here for file

Additional file 3 **Slight unfolding of ANKS6-SAM R823W observed by SEC-MALS.** ANKS6-SAM containing the R823W mutation is slightly unfolded compared to ANKS6-SAM wt, as evidenced by the faster migration on SEC-MALS and the slightly increased molecular mass: 8.4 kDa for ANKS6-SAM R823W versus 7.8 kDa for ANKS6-SAM wt. This apparent increase in molecular mass is consistent with a protein that is partially unfolded and therefore exhibits a larger radius of gyration. Click here for file
