# Supplementary material for: Characterization of the SAM domain of the PKD-related protein ANKS6 and its interaction with ANKS3
Source: BMC Struct Biol. 2014 Jul 7;14:17. doi: 10.1186/1472-6807-14-17 (PMC4105859; doi:10.1186/1472-6807-14-17)
Supplement: Additional file 1 — Human SAM domains screened for interaction with ANKS3-SAM. negGFP-SAM-domain fusions of the following human SAM-domain containing proteins were screened for binding to negGFP-ANKS3-SAM using the negGFP native gel assay. Sequences of SAM domains used and cloning are as described previously [22]. Where “2SAMs” is listed, the construct contains two SAM domains in tandem. [file 1472-6807-14-17-S1.pdf]

Human SAM-domain containing protein	UNIPROT ID	Residues
AIDA1C/ANKS1B/ cajalin- 2SAMs	Q7Z6G8	808-950
ANKS1A/ odin- 2SAMs	Q92625	692-836
ANKS6	Q68DC2	771-840
ARAP1/CentaurinD2	Q96P48	1-70
ARAP2/Centaurin D1	Q8WZ64	1-71
BAR	Q9NZS9	177-252
C14orf174	Q9P1V8	541-610
CNKSR2	Q8WXI2	1-77
CNKSR3	Q6P9H4	1-73
DDHD-containing 2	O94830	382-449
ELF3	P78545	48-135
ELF5	Q9UKW6	44-129
EPHA2	P29317	899-970
EPHA5	P54756	960-1030
EPHA6	Q9UF33	956-1023
EPHA7	Q15375	919-998
EPHB1	P54762	906-984
EPHB2	P29323	906-985
EPHB6	O15197	945-1021
ESE3	Q9NZC4	41-120
ETS1	P14921	53-136
GA-binding protein/GABPA	Q06546	170-251
INPPL1	O15357	194-1258
LRSAM1	Q6UWE0	564-634
MOB	Q86VZ5	7-78
Neurabin-1	Q9ULJ8	983-1054
SAMD12	Q8N8I0	71-145
SAMD4B	Q5PRF9	296-361
SAMHD1	Q9Y3Z3	1-111
SAMSN1	Q9NSI8	237-306
SASH1-SAM1	O94885	625-699
SASH1-SAM2	O94885	1173-1247
SASH3	O75995	247-318
SEC23-interacting protein/SEC23IP	Q9Y6Y8	642-703
SLP76/LCP2	Q13094	10-80
Smaug/SAMD4A	Q9UPU9	320-383
StarD13-deletion	Q9Y3MB	154-241
STIM2	Q9P246	129-205
TP63	Q9H3D4	541-609
Usher1G-optimized	Q495M9	381-461
WDSUB1	Q8N9V3	329-396
