# Supplementary figures and images for: Characterization of the SAM domain of the PKD-related protein ANKS6 and its interaction with ANKS3
Source: BMC Struct Biol. 2014 Jul 7;14:17. doi: 10.1186/1472-6807-14-17 (PMC4105859; doi:10.1186/1472-6807-14-17)

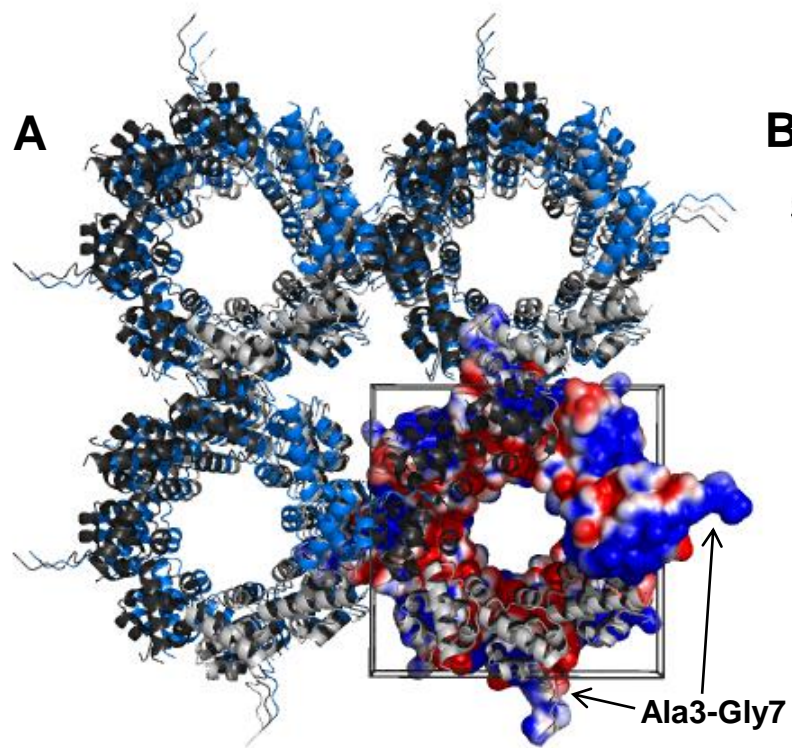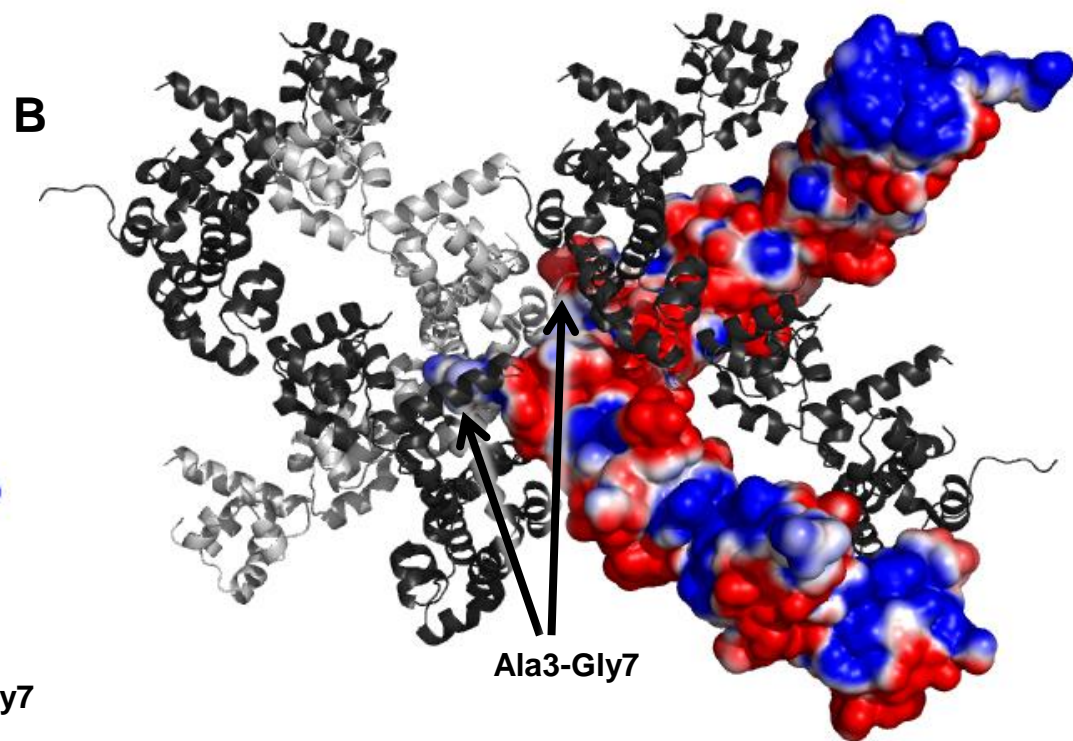

Supplement: Additional file 2 — Crystal packing of ANKS3-SAM triple helices. A) Individual ANKS3-SAM polymers intertwine to create a triple helix. Triple helices pack side-by-side in the crystal structure. A single ANKS3-SAM triple helix fills the unit cell, shown as a boxed outline. Individual polymers in each triple helix are colored blue, grey, and black. A single polymer is shown as a space-filled model colored by surface electrostatics generated using APBS in Pymol and contouring at ± 1kT/e. Within a single ANKS3-SAM polymer, the N-terminal arm (residues Ala3-Gly7) extends outward from every other SAM domain in the helical segment and forms contacts with a neighboring polymer of triple helices. B) Closer view of the N-terminal arm swapping that occurs between polymers. Two polymers of triple helices are shown, colored as above. Within each, a single polymer has been removed for clarity. Residues Ala3-Gly7 intercalate between two polymers of an adjacent triple helix, thereby forming crystal contacts which stabilize the observed triple helix. [file 1472-6807-14-17-S2.pdf]

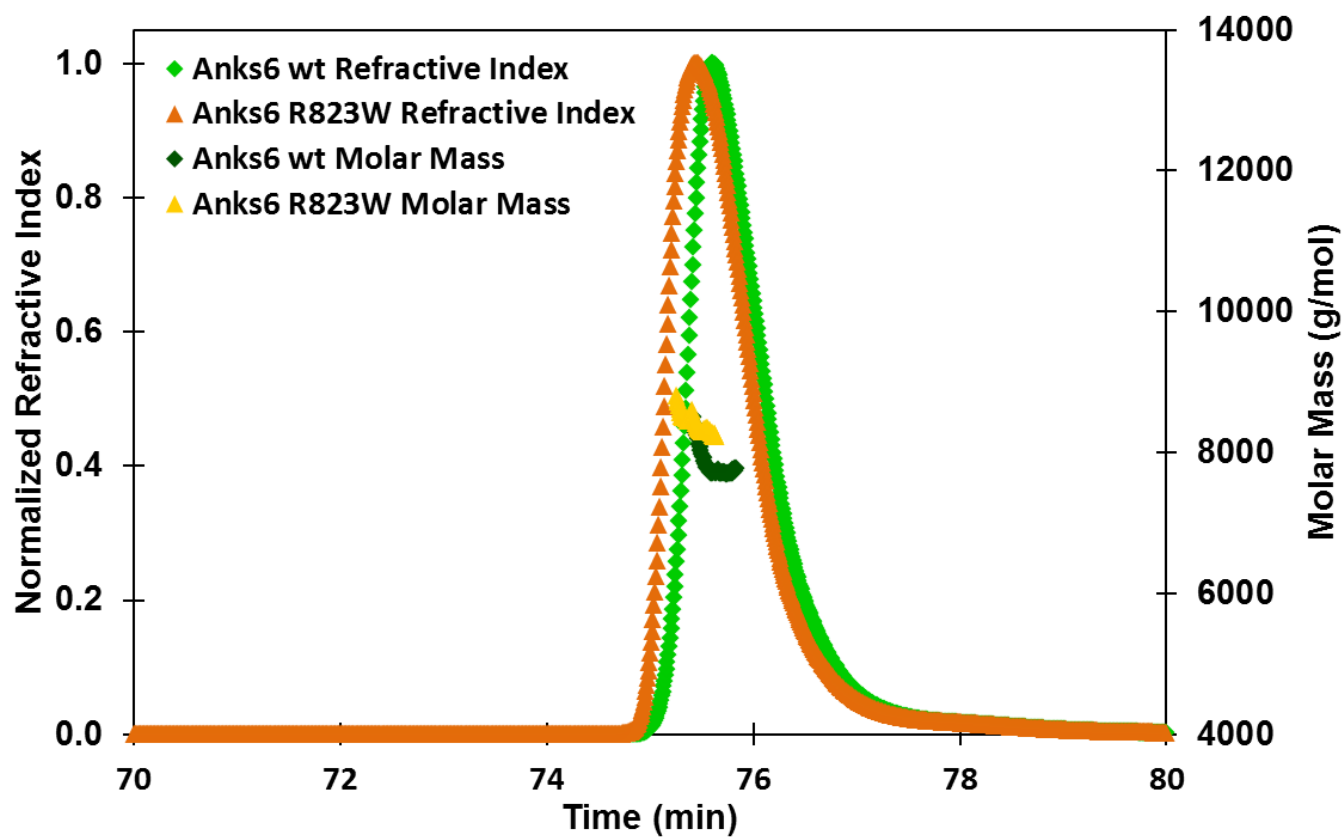

Supplement: Additional file 3 — Slight unfolding of ANKS6-SAM R823W observed by SEC-MALS. ANKS6-SAM containing the R823W mutation is slightly unfolded compared to ANKS6-SAM wt, as evidenced by the faster migration on SEC-MALS and the slightly increased molecular mass: 8.4 kDa for ANKS6-SAM R823W versus 7.8 kDa for ANKS6-SAM wt. This apparent increase in molecular mass is consistent with a protein that is partially unfolded and therefore exhibits a larger radius of gyration. [file 1472-6807-14-17-S3.pdf]
